# A cyclo­octa­trienone complex of diiron hexa­carbon­yl

**DOI:** 10.1107/S1600536814012690

**Published:** 2014-06-07

**Authors:** Peter W. R. Corfield

**Affiliations:** a Department of Chemistry, Fordham University, 441 East Fordham Road, Bronx, NY 10458, USA

## Abstract

In the title compound, [μ-(2,6,7-η:3,4,5-η)-cycloocta-2,4,6-trienone]bis­(tri­carbonyl­iron)(*Fe*—*Fe*), [Fe_2_(C_8_H_8_O)(CO)_6_], the diiron hexa­carbonyl moiety has a sawhorse arrangement, with the OC—Fe—Fe—CO fragment forming the horizontal bar of the horse, and the other four carbonyl groups the legs. The Fe—Fe distance is 2.795 (2) Å. Each Fe atom is also bonded to three C atoms of the cyclo­octa­trienone ring. One Fe atom forms a σ-bond with one ring C atom, with Fe—C = 2.109 (2) Å, and also a metal–olefin π-bond with two C atoms on the other side of the ring, with Fe—C distances of 2.238 (2) and 2.236 (3) Å. The second Fe atom forms a η^3^-allyl bond with three other ring atoms, with Fe—C bond lengths of 2.158 (2), 2.062 (2), and 2.123 (3) Å. Counting the π- and π-allyl inter­actions as one bond, the coordinations of the Fe atoms can, respectively, be approximated as octa­hedral and trigonal bipyramidal.

## Related literature   

The title compound was synthesized as part of a study on reactions of various cyclo­octa­tetra­ene iron carbonyls (Paquette *et al.*, 1975 ▶). The first reported synthesis of the compound was by King (1963 ▶). The structure of the corresponding cyclo­octa­triene complex was reported by Cotton & Edwards (1969 ▶), and that of a closely related derivative by Kerber *et al.* (1984 ▶), who also review other related structures.
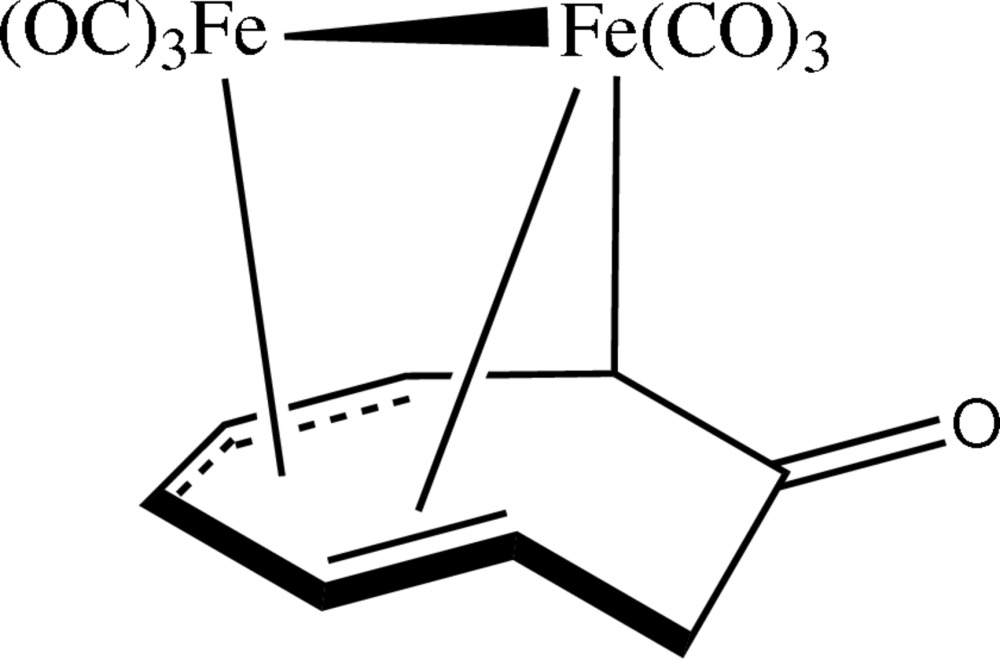


## Experimental   

### 

#### Crystal data  

[Fe_2_(C_8_H_8_O)(CO)_6_]
*M* *_r_* = 399.90
Triclinic, 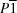

*a* = 7.729 (8) Å
*b* = 8.258 (8) Å
*c* = 11.927 (11) Å
α = 89.172 (16)°
β = 83.82 (3)°
γ = 74.54 (2)°
*V* = 729.4 (12) Å^3^
*Z* = 2
Mo *K*α radiation
μ = 2.02 mm^−1^
*T* = 296 K
0.5 × 0.4 × 0.3 mm

#### Data collection  

Picker four-circle diffractometer
Absorption correction: integration (Busing & Levy, 1957 ▶) *T*_min_ = 0.48, *T*_max_ = 0.58
4520 measured reflections
4227 independent reflections
3687 reflections with *I* > 2σ(*I*)
*R*_int_ = 0.019
18 standard reflections every 500 reflections intensity decay: 7.6(1)

#### Refinement  

*R*[*F* ^2^ > 2σ(*F* ^2^)] = 0.027
*wR*(*F* ^2^) = 0.091
*S* = 1.11
4226 reflections
208 parameters
H-atom parameters constrained
Δρ_max_ = 0.34 e Å^−3^
Δρ_min_ = −0.49 e Å^−3^

### 

Data collection: Corfield (1972 ▶); cell refinement: Corfield (1972 ▶); data reduction: data reduction followed procedures in Corfield *et al.* (1973 ▶), with *p* = 0.06 [data were averaged with a local version of *SORTAV* (Blessing, 1989 ▶), and a four-dimensional scaling procedure (*XABS2*; Parkin *et al.*, 1995 ▶) was applied]; program(s) used to solve structure: local superposition program (Corfield, 1972 ▶); program(s) used to refine structure: *SHELXL97* (Sheldrick, 2008 ▶); molecular graphics: *ORTEPIII* (Burnett & Johnson, 1996 ▶); software used to prepare material for publication: *SHELXL97*.

## Supplementary Material

Crystal structure: contains datablock(s) I. DOI: 10.1107/S1600536814012690/pk2527sup1.cif

Structure factors: contains datablock(s) I. DOI: 10.1107/S1600536814012690/pk2527Isup2.hkl

CCDC reference: 1006076

Additional supporting information:  crystallographic information; 3D view; checkCIF report

## Figures and Tables

**Fig. 1.**
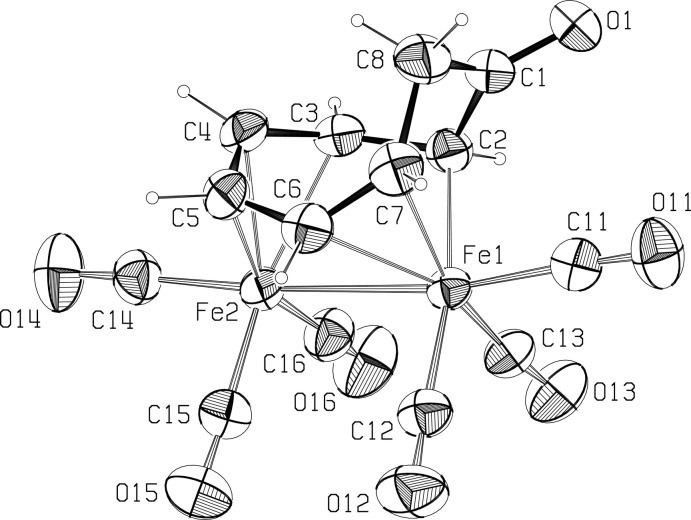
The molecular structure of the title molecule, with ellipsoids at the 50% level.

**Fig. 2.**
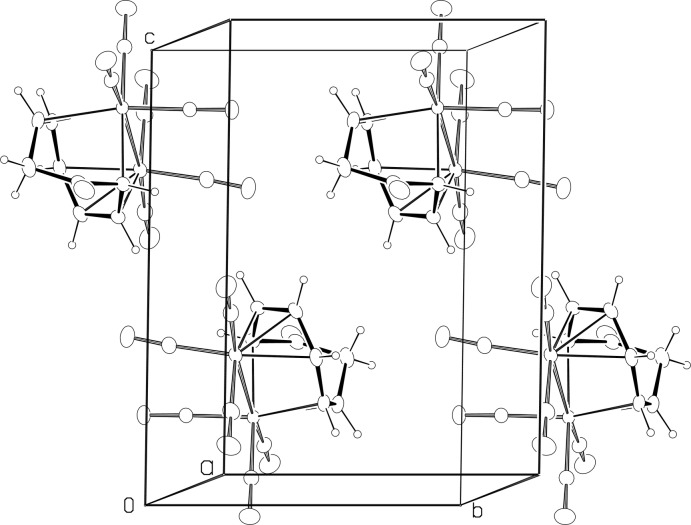
Packing of the title complex, viewed along the a* axis, with ellipsoid outlines at 30% probability.

## supplementary crystallographic information

### S1.1. Synthesis and crystallization

Details of the synthesis of the title compound are given in Paquette *et al.* (1975), which describes how the structure relates to mechanistic studies on cyclo­additions of substituted cyclo­octa­tetra­enes to iron carbonyl complexes. A previous synthesis by a different route is given by King (1963).

### S1.2. Refinement

Each of the 18 standard reflections was measured 17-18 times during the 97 hours of data collection. Decay of individual standards during data collection was relatively isotropic, ranging from 6.9 (2)%-8.3 (5)%. Data were collected in two shells, θ=0-20° and θ=20-30°. The average decay during collection of data in the first shell was 0.8 (1)%, so that most of the decay occurred during collection of weaker intensities in the higher angle shell. No correction was made for the fall-off in standard intensities.

The original data reduction deleted 536 reflections with I<2σ(I)out of a total of 4226 measurements, and their details are no longer available. Near the end of the final refinements, the missing reflections were reinserted into the data file, with F^2^ values set equal to the σ(F^2^) found for reflections with F^2^<3σ(F^2^), averaged over ten ranges of theta values.

This report is based upon refinements that include the reinserted weaker reflections. The necessarily arbitrary assignment of F^2^ values for these reflections with I<2σ(I) is the reason for the high K value for the weakest reflections in the final refinement. The structure was also refined without these weak reflections. The average Δ/σ for all parameters between refinements with and without the missing reflections was 0.21, with a maximum of 0.67 for U_11_ for Fe1.

One reflection, (-2, 1, 3), was omitted from the final refinements, as the records clearly indicate an error during the scan. As noted by the checkCIF/ PLATON report, alert level B, there are two reflections which show large Δ(F^2^)/σ values in the final refinements, (-2, 0, 2) and (0, 1, 5). The calculated F^2^ values for these weaker reflections are near zero. However, as the chart record clearly shows peaks during these scans, there seemed no reason to delete them from the reflections file.

Positions of the two Fe atoms and the atoms of the six carbonyl groups were found by superposition methods.

H atoms were constrained to idealized positions with C—H distances of 0.98Å for the tertiary H atoms on C2—7 and 0.97Å for the secondary H atoms on C8. The U_eq_ values for all H atoms were fixed at 1.2 times the U_iso_ of their bonded C atoms.

Initial refinements with anisotropic temperature factors for Fe, O and C atoms and constrained hydrogen atom parameters converged smoothly, but a difference Fourier synthesis at this stage showed a pattern of peaks and holes 0.8-0.9 Å from the iron atoms, with maximum and minimum density values of 0.66 and -0.42 e/A^3^. The intensity data were smoothed by a 12 parameter model with *XABS2* (Parkin *et al.*, 1995), to allow for systematic anisotropies that might have existed in the data collection. After ensuing refinements, the maximum and minimum residual electron densities were reduced to 0.34 and -0.49 e/A^3^.

### S2. Comment

The title compound was synthesized as part of a study on the mechanism of cyclo­addition of tetra­cyano­ethyl­ene to various cyclo­octa­tetra­ene iron carbonyls. (Paquette *et al.*, 1975). Determination of this structure clearly showed that Fe1 was σ bonded to C2 and π-bonded to C6 and C7, distinguishing the compound from a possible isomer with Fe1 σ bonded to C7 and π-bonded to the other side of the ring.

The structure of the corresponding cyclo­octa­triene complex was reported by Cotton and Edwards (1969), and that of a related carboxyl­ate derivative by reported by Kerber *et al.* (1984), who also review two other related structures involving a bi­cyclo arrangement at C8 and C1.

In the present compound, the di-iron hexa­carbonyl moiety has a sawhorse arrangement, with the fragment O11—C11—Fe1—Fe2—C14—O14 forming the horizontal bar of the horse, and the other four carbonyl groups the legs. The Fe—Fe distance is 2.795 (2) Å, somewhat longer than in similar structures reviewed by Kerber *et al.* (1984), in which the Fe—Fe distances range from 2.764 (3)Å to 2.786 (2)Å.

Each Fe atom is also bonded to three of the cyclo­octa­trienone carbon atoms. Fe1 forms a σ-bond with C2, with Fe—C2 = 2.109 (2)Å, and a metal-olefin π-bond with C6 and C7, with Fe1—C6 = 2.238 (2) Å and Fe1—C7 = 2.236 (3) Å. Counting the π-coordination as one bond, Fe1 can be considered as o­cta­hedrally coordinated. The shorter σ-bond length and the longer distances to the π-bonded C atoms are similar to those reported by Kerber *et al.* (1984).

Fe2 forms a *trihapto*allyl bond with atoms C3—C5, with bond distances Fe2—C3 = 2.158 (2) Å, Fe2—C4 = 2.062 (2) Å and Fe2—C5 = 2.123 (3) Å. Counting the π-allyl inter­action as one bond, the coordination of Fe2 can be approximated as trigonal bipyramidal. The irregular pattern of distances lying in between those for the σ- and π-bonds is again similar to those found previously (Kerber *et al.*, 1984).

Four of the Fe—C carbonyl distances are clustered closely around a mean of 1.800 (2)Å. However, Fe1—C12 is somewhat longer, at 1.818 (2), and Fe1—C13 is somewhat shorter, at 1.783 (2)Å, perhaps reflecting the *trans* positions of these bonds to the Fe1—C2 σ bond and the Fe1—C6,C7 π bond, respectively.

The cyclo­octa­trienone ring is buckled in a complex way due to the trihapto bonding to each of the two Fe atoms. One might expect the six bonded C atoms to be held closer to the Fe atoms and the remaining two ring C atoms to be bent away from the Fe atoms, and this indeed appears to be the case. The bonded ring atoms C2—C7 form a rough plane, with rms deviation of 0.26 Å, and atoms C8 and C1 are displaced 1.543 (3)Å and 1.002 (3)Å respectively from this plane, away from the Fe atoms. Distances and angles for the ring are given in Table 1.

The shortest inter­molecular contact is H4—H4(1-x,1-y,1-z), at 2.39Å. The shortest inter­molecular distance between carbonyl groups is O4—O4 (2-x,-y,1-z) at 3.042 (4)Å. Contacts between hydrogen atoms and oxygen atoms range upwards from 2.81Å, for O14—H3(-x,1-y,-z).

### Crystal data

**Table:**

[Fe_2_(C_8_H_8_O)(CO)_6_]	*Z* = 2
*M**_r_* = 399.90	*F*(000) = 400
Triclinic, *P*1	*D*_x_ = 1.821 Mg m^−^^3^*D*_m_ = 1.83 Mg m^−^^3^*D*_m_ measured by flotation in bromobenzene/bromoform mixture
Hall symbol: -P 1	Melting point: 428 K
*a* = 7.729 (8) Å	Mo *K*α radiation, λ = 0.71070 Å
*b* = 8.258 (8) Å	Cell parameters from 12 reflections
*c* = 11.927 (11) Å	θ = 11.0–25.5°
α = 89.172 (16)°	µ = 2.02 mm^−^^1^
β = 83.82 (3)°	*T* = 296 K
γ = 74.54 (2)°	Block, red
*V* = 729.4 (12) Å^3^	0.5 × 0.4 × 0.3 mm

### Data collection

**Table:**

Picker four-circle diffractometer	3687 reflections with *I* > 2σ(*I*)
Radiation source: sealed X-ray tube	*R*_int_ = 0.019
Oriented graphite 200 reflection monochromator	θ_max_ = 29.9°, θ_min_ = 2.6°
θ/2θ scans	*h* = −10→10
Absorption correction: integration (Busing & Levy, 1957)	*k* = −11→11
*T*_min_ = 0.48, *T*_max_ = 0.58	*l* = 0→16
4520 measured reflections	18 standard reflections every 500 reflections
4227 independent reflections	intensity decay: 7.6(1)

### Refinement

**Table:**

Refinement on *F*^2^	Primary atom site location: heavy-atom method
Least-squares matrix: full	Secondary atom site location: difference Fourier map
*R*[*F*^2^ > 2σ(*F*^2^)] = 0.027	Hydrogen site location: inferred from neighbouring sites
*wR*(*F*^2^) = 0.091	H-atom parameters constrained
*S* = 1.11	*w* = 1/[σ^2^(*F*_o_^2^) + 0.084*P*] where *P* = (*F*_o_^2^ + 2*F*_c_^2^)/3
4226 reflections	(Δ/σ)_max_ < 0.001
208 parameters	Δρ_max_ = 0.34 e Å^−^^3^
0 restraints	Δρ_min_ = −0.49 e Å^−^^3^

### Special details

**Table:**

Geometry. All e.s.d.'s (except the e.s.d. in the dihedral angle between two l.s. planes) are estimated using the full covariance matrix. The cell e.s.d.'s are taken into account individually in the estimation of e.s.d.'s in distances, angles and torsion angles; correlations between e.s.d.'s in cell parameters are only used when they are defined by crystal symmetry. An approximate (isotropic) treatment of cell e.s.d.'s is used for estimating e.s.d.'s involving l.s. planes.
Refinement. Refinement of *F*^2^ against ALL reflections. The weighted *R*-factor *wR* and goodness of fit *S* are based on *F*^2^, conventional *R*-factors *R* are based on *F*, with *F* set to zero for negative *F*^2^. The threshold expression of *F*^2^ > σ(*F*^2^) is used only for calculating *R*-factors(gt) *etc*. and is not relevant to the choice of reflections for refinement. *R*-factors based on *F*^2^ are statistically about twice as large as those based on *F*, and *R*-factors based on ALL data will be even larger.

### Fractional atomic coordinates and isotropic or equivalent isotropic displacement parameters (Å^2^)

**Table:**

	*x*	*y*	*z*	*U*_iso_*/*U*_eq_	
Fe1	0.23879 (3)	0.27972 (3)	0.177172 (18)	0.03093 (8)	
Fe2	0.53855 (3)	0.14576 (3)	0.29382 (2)	0.03417 (8)	
C11	0.0206 (3)	0.3694 (2)	0.13055 (15)	0.0413 (4)	
O11	−0.1162 (2)	0.4219 (2)	0.09807 (15)	0.0624 (4)	
C12	0.3569 (3)	0.2465 (2)	0.03568 (15)	0.0432 (4)	
O12	0.4271 (3)	0.2256 (2)	−0.05374 (13)	0.0677 (5)	
C13	0.2055 (3)	0.0741 (2)	0.18355 (15)	0.0394 (3)	
O13	0.1684 (3)	−0.05060 (18)	0.18661 (14)	0.0580 (4)	
C14	0.7292 (3)	0.0818 (2)	0.37346 (17)	0.0450 (4)	
O14	0.8500 (2)	0.0358 (2)	0.42302 (17)	0.0682 (5)	
C15	0.6776 (3)	0.1002 (3)	0.16087 (18)	0.0483 (4)	
O15	0.7697 (3)	0.0716 (3)	0.07895 (15)	0.0752 (5)	
C16	0.4823 (3)	−0.0514 (2)	0.31890 (17)	0.0442 (4)	
O16	0.4551 (3)	−0.17724 (19)	0.33981 (16)	0.0674 (5)	
C1	0.0240 (2)	0.4664 (2)	0.35754 (15)	0.0414 (4)	
O1	−0.13837 (19)	0.4908 (2)	0.38358 (13)	0.0571 (4)	
C2	0.1512 (2)	0.2959 (2)	0.35132 (13)	0.0361 (3)	
H2	0.0868	0.2108	0.3725	0.043*	
C3	0.3129 (2)	0.2703 (2)	0.41251 (14)	0.0372 (3)	
H3	0.3051	0.2147	0.4853	0.045*	
C4	0.4485 (2)	0.3564 (2)	0.39688 (15)	0.0409 (4)	
H4	0.5158	0.3642	0.4608	0.049*	
C5	0.5108 (3)	0.4085 (2)	0.29046 (17)	0.0421 (4)	
H5	0.6234	0.4426	0.2868	0.051*	
C6	0.4023 (3)	0.4619 (2)	0.19756 (16)	0.0404 (4)	
H6	0.4715	0.4828	0.1277	0.049*	
C7	0.2215 (3)	0.5506 (2)	0.20695 (17)	0.0437 (4)	
H7	0.1860	0.6237	0.1432	0.052*	
C8	0.1071 (3)	0.6065 (2)	0.31726 (19)	0.0508 (5)	
H8A	0.0130	0.7082	0.3068	0.061*	
H8B	0.1809	0.6296	0.3726	0.061*	

### Atomic displacement parameters (Å^2^)

**Table:**

	*U*^11^	*U*^22^	*U*^33^	*U*^12^	*U*^13^	*U*^23^
Fe1	0.03534 (13)	0.02750 (12)	0.03108 (12)	−0.01090 (9)	−0.00212 (9)	0.00032 (8)
Fe2	0.03353 (13)	0.03090 (12)	0.03838 (13)	−0.00883 (9)	−0.00396 (9)	−0.00376 (9)
C11	0.0447 (9)	0.0389 (8)	0.0413 (8)	−0.0132 (7)	−0.0048 (7)	0.0058 (7)
O11	0.0474 (8)	0.0741 (10)	0.0661 (10)	−0.0131 (8)	−0.0181 (7)	0.0176 (8)
C12	0.0504 (10)	0.0408 (9)	0.0391 (8)	−0.0149 (8)	−0.0010 (7)	−0.0007 (7)
O12	0.0817 (12)	0.0784 (11)	0.0412 (8)	−0.0256 (10)	0.0131 (8)	−0.0061 (7)
C13	0.0477 (9)	0.0348 (8)	0.0381 (8)	−0.0141 (7)	−0.0076 (7)	0.0007 (6)
O13	0.0793 (11)	0.0389 (7)	0.0653 (9)	−0.0293 (7)	−0.0165 (8)	0.0043 (6)
C14	0.0395 (9)	0.0419 (9)	0.0551 (10)	−0.0130 (7)	−0.0060 (8)	0.0007 (8)
O14	0.0513 (10)	0.0718 (10)	0.0879 (12)	−0.0195 (8)	−0.0297 (9)	0.0192 (9)
C15	0.0438 (10)	0.0486 (10)	0.0507 (10)	−0.0101 (8)	−0.0020 (8)	−0.0077 (8)
O15	0.0625 (11)	0.0987 (14)	0.0587 (10)	−0.0190 (10)	0.0148 (8)	−0.0195 (9)
C16	0.0459 (10)	0.0375 (8)	0.0503 (10)	−0.0102 (7)	−0.0125 (8)	0.0004 (7)
O16	0.0865 (13)	0.0418 (8)	0.0838 (12)	−0.0274 (8)	−0.0280 (10)	0.0138 (8)
C1	0.0388 (9)	0.0446 (9)	0.0384 (8)	−0.0081 (7)	−0.0005 (7)	−0.0094 (7)
O1	0.0356 (7)	0.0722 (10)	0.0586 (8)	−0.0081 (7)	0.0032 (6)	−0.0170 (7)
C2	0.0374 (8)	0.0370 (8)	0.0353 (7)	−0.0150 (6)	0.0027 (6)	−0.0035 (6)
C3	0.0437 (9)	0.0357 (7)	0.0316 (7)	−0.0105 (7)	−0.0006 (6)	−0.0048 (6)
C4	0.0435 (9)	0.0353 (8)	0.0448 (9)	−0.0098 (7)	−0.0085 (7)	−0.0113 (7)
C5	0.0401 (9)	0.0331 (8)	0.0571 (10)	−0.0175 (7)	−0.0025 (8)	−0.0062 (7)
C6	0.0472 (9)	0.0307 (7)	0.0469 (9)	−0.0193 (7)	0.0021 (7)	0.0014 (6)
C7	0.0512 (10)	0.0284 (7)	0.0532 (10)	−0.0138 (7)	−0.0057 (8)	0.0046 (7)
C8	0.0489 (11)	0.0313 (8)	0.0674 (12)	−0.0037 (7)	−0.0018 (9)	−0.0107 (8)

### Geometric parameters (Å, º)

**Table:**

Fe1—C13	1.783 (2)	C16—O16	1.133 (3)
Fe1—C11	1.796 (2)	C1—O1	1.223 (3)
Fe1—C12	1.818 (2)	C1—C2	1.484 (3)
Fe1—C2	2.109 (2)	C2—C3	1.480 (3)
Fe1—C7	2.236 (3)	C2—H2	0.9800
Fe1—C6	2.238 (2)	C3—C4	1.411 (3)
Fe1—Fe2	2.795 (2)	C3—H3	0.9800
Fe2—C14	1.795 (2)	C4—C5	1.411 (3)
Fe2—C15	1.802 (3)	C4—H4	0.9800
Fe2—C16	1.807 (2)	C5—C6	1.454 (3)
Fe2—C4	2.062 (2)	C5—H5	0.9800
Fe2—C5	2.123 (3)	C6—C7	1.387 (3)
Fe2—C3	2.158 (2)	C6—H6	0.9800
C11—O11	1.138 (3)	C7—C8	1.508 (3)
C12—O12	1.137 (3)	C7—H7	0.9800
C13—O13	1.140 (2)	C8—H8A	0.9700
C14—O14	1.135 (3)	C8—H8B	0.9700
O14—O14^i^	3.042 (4)	C8—C1	1.515 (3)
C15—O15	1.133 (3)		
			
C13—Fe1—C11	92.47 (9)	O15—C15—Fe2	177.8 (2)
C13—Fe1—C12	93.48 (9)	O16—C16—Fe2	175.41 (18)
C11—Fe1—C12	94.65 (10)	O1—C1—C2	122.70 (18)
C13—Fe1—C2	85.60 (8)	O1—C1—C8	122.10 (18)
C11—Fe1—C2	96.41 (9)	C2—C1—C8	114.90 (17)
C12—Fe1—C2	168.93 (8)	C3—C2—C1	117.09 (15)
C13—Fe1—C7	164.71 (8)	C3—C2—Fe1	107.62 (12)
C11—Fe1—C7	81.66 (8)	C1—C2—Fe1	99.86 (11)
C12—Fe1—C7	101.03 (8)	C3—C2—H2	110.6
C2—Fe1—C7	81.08 (7)	C1—C2—H2	110.6
C13—Fe1—C6	152.39 (8)	Fe1—C2—H2	110.6
C11—Fe1—C6	115.14 (10)	C4—C3—C2	127.37 (16)
C12—Fe1—C6	84.91 (9)	C4—C3—Fe2	66.83 (11)
C2—Fe1—C6	90.79 (7)	C2—C3—Fe2	105.68 (12)
C7—Fe1—C6	36.13 (8)	C4—C3—H3	114.9
C13—Fe1—Fe2	85.85 (7)	C2—C3—H3	114.9
C11—Fe1—Fe2	168.17 (6)	Fe2—C3—H3	114.9
C12—Fe1—Fe2	97.13 (9)	C5—C4—C3	123.23 (16)
C2—Fe1—Fe2	71.80 (7)	C5—C4—Fe2	72.62 (12)
C7—Fe1—Fe2	97.01 (6)	C3—C4—Fe2	74.20 (11)
C6—Fe1—Fe2	67.10 (8)	C5—C4—H4	118.0
C14—Fe2—C15	92.80 (12)	C3—C4—H4	118.0
C14—Fe2—C16	91.69 (9)	Fe2—C4—H4	118.0
C15—Fe2—C16	100.74 (10)	C4—C5—C6	125.64 (18)
C14—Fe2—C4	87.79 (9)	C4—C5—Fe2	68.01 (10)
C15—Fe2—C4	133.75 (9)	C6—C5—Fe2	102.46 (11)
C16—Fe2—C4	125.48 (10)	C4—C5—H5	115.9
C14—Fe2—C5	99.31 (8)	C6—C5—H5	115.9
C15—Fe2—C5	95.38 (9)	Fe2—C5—H5	115.9
C16—Fe2—C5	160.00 (9)	C7—C6—C5	126.10 (18)
C4—Fe2—C5	39.37 (8)	C7—C6—Fe1	71.86 (12)
C14—Fe2—C3	106.02 (11)	C5—C6—Fe1	108.79 (12)
C15—Fe2—C3	158.05 (9)	C7—C6—H6	113.9
C16—Fe2—C3	90.10 (10)	C5—C6—H6	113.9
C4—Fe2—C3	38.97 (7)	Fe1—C6—H6	113.9
C5—Fe2—C3	70.88 (8)	C6—C7—C8	124.40 (19)
C14—Fe2—Fe1	174.00 (6)	C6—C7—Fe1	72.01 (10)
C15—Fe2—Fe1	89.25 (10)	C8—C7—Fe1	108.43 (12)
C16—Fe2—Fe1	93.46 (8)	C6—C7—H7	114.6
C4—Fe2—Fe1	86.73 (7)	C8—C7—H7	114.6
C5—Fe2—Fe1	74.87 (6)	Fe1—C7—H7	114.6
C3—Fe2—Fe1	70.93 (8)	C7—C8—C1	107.95 (15)
O11—C11—Fe1	177.54 (19)	C7—C8—H8A	110.1
O12—C12—Fe1	178.4 (2)	C1—C8—H8A	110.1
O13—C13—Fe1	173.85 (19)	C7—C8—H8B	110.1
O14—C14—Fe2	177.66 (19)	C1—C8—H8B	110.1
C14—O14—O14^i^	170.9 (2)	H8A—C8—H8B	108.4
			
C1—C2—C3—C4	−58.8 (2)	C6—C7—C8—C1	−88.8 (2)
C2—C3—C4—C5	−36.3 (3)	C7—C8—C1—C2	46.3 (2)
C3—C4—C5—C6	32.9 (3)	C8—C1—C2—C3	57.0 (2)
C4—C5—C6—C7	34.9 (3)	O1—C1—C2—C3	−129.21 (19)
C5—C6—C7—C8	0.0 (3)	O1—C1—C8—C7	−127.55 (19)

Symmetry code: (i) −*x*+2, −*y*, −*z*+1.
